# Effect of different biofloc starters on ammonia, nitrate, and nitrite concentrations in the cultured tilapia *Oreochromis niloticus *system

**DOI:** 10.12688/f1000research.22977.3

**Published:** 2020-06-02

**Authors:** Iskandar Putra, Irwan Effendi, Iesje Lukistyowati, Usman M. Tang, Muhammad Fauzi, Indra Suharman, Zainal A. Muchlisin

**Affiliations:** 1 Faculty of Fisheries and Marine Science, Universitas Riau, Pekanbaru, Riau, 28000, Indonesia; 2 Faculty of Marine and Fisheries, Universitas Syiah Kuala, Banda Aceh, Indonesia

**Keywords:** Biofloc, Carbon, Molasses, Water Quality

## Abstract

**Background: **High stocking density and intensive feeding in aquaculture systems lead to the accumulation of organic waste, which results in an increase in ammonia, nitrite, and nitrite concentrations in culture media. Biofloc is a potential technology to overcome this problem. The starter is a crucial carbon source for bacteria in the formation of biofloc. The objective of the present study aimed to explore the best starter of biofloc in a red tilapia *Oreochromis niloticus* culture system.

**Methods: **A completely randomized design with four levels of treatment was used in this study. The tested starter was (A) control treatment, biofloc without starter, (B) biofloc with molasses starter, (C) biofloc with tapioca starter, and (D) biofloc with sucrose starter. The floc was cultured in 100-L tanks with a salinity of 17 ppt. The tanks were stocked with *O.* *niloticus* with a size of 3.71±0.11 cm at a stocking density of 30 fish per tank. The fish were fed on a commercial diet two times a day at satiation for 40 days. The ammonia, nitrite, and nitrite concentrations were measured for an interval of 8 days.

**Results: **The study showed that the NH _3_-N range was 0.02–0.07 mg L ^−1^ (mean, 0.03 ± 0.02 mg L ^−1^), NO _2_-N range was 0.20–0.43 mg L ^−1^ (mean, 0.25 ± 0.12 mg L ^−1^), and NO _3_-N range was 0.90–3.20 mg L ^−1^ (mean, 1.42 ± 1.19 mg L ^−1^).

**Conclusion: **Among the starters tested, molasses was found to be the best for biofloc in tilapia culture.

## Introduction

Water quality is a crucial factor in aquaculture systems. One important water quality parameter is nitrogen. In water, nitrogen can be found in the forms of ammonia, nitrite, and nitrate ^1– 5^. Ammonia (NH _3_-N) is produced from the breakdown of proteins from unconsumed feed, feces, and urine of fish. This compound will turn into nitrite (NO _2_) when oxygen levels are poor ^6^, which is toxic for fish ^7^. By contrast, ammonia is changed into nitrate when the dissolved oxygen level is sufficient ^8, 9^. Fish produces ammonia (inorganic N) through the osmoregulation process; feces and urine contribute about 10%–20% of total nitrogen ^10^. The application of biofloc is one of the alternatives to overcome water quality problems especially in controlling total ammonia nitrogen in the aquaculture system ^11– 13^.

Biofloc refers to the use of heterotrophs and autotrophs, which can convert organic waste into floc forms that can be utilized by fish as a food source ^4, 11, 14– 16^. Biofloc technology is cheap, simple, and environmentally friendly ^13, 17^. Several organisms, such as bacteria, plankton, fungi, and algae, and suspended particles exist in flocs ^18, 19^. These organisms provide nutrition for cultured fish. However, the formation of biofloc needs a starter consisting of probiotics and a carbon source. Molasses, tapioca, and wheat flours are common starters in biofloc culture (African catfish, Nile tilapia, *Litopenaeus vannamei*) ^16, 19– 23^. Presently, limited information is available on the best starter for biofloc in the cultured system of red tilapia *Oreochromis niloticus*. Thus, the present study aimed to explore the best starter for biofloc in a red tilapia *O. niloticus* culture system.

## Methods

### Time and site

The research was carried out for 40 days from February 2019 to March 2019 at the Aquaculture Technology Laboratory, Faculty of Fisheries and Marine, Riau University, Indonesia. The experiments were carried out within the ethical guidelines in animal research developed by NC3Rs. In Indonesia, no approval is required to conduct research on fish.

### Experimental design

A completely randomized design with four levels of treatment and three replications was performed in this study; the tested treatment was four starters of biofloc, namely control without a starter (treatment A), biofloc with molasses starter concentration 0.48 gL ^−1^ (treatment B), biofloc with tapioca starter concentration 0.35 gL ^−1^ (treatment C), and biofloc with sucrose concentration 0.42 gL ^−1^ (treatment D).

The amount of carbon added is calculated based on the carbon content (C) in the ingredients and the nitrogen content in the feed given ^5^ using the formula:

C/N = (% C starter x molecule weight of starter + % C feed x feed weight)/ % N feed x feed weight. Based on this formula, to obtain a C/N ratio of 20: 1, a carbon source derived from molasses was 48 g with a C content of 37% ^24^, tapioca flour as much as 35 g with a C content of 50.3% ^25^, and sucrose 42 g with a C content of 42.3% ^26^.

### Biofloc culture

Biofloc was cultured using 0.01 ml/L probiotic bacteria from Boster Multisel ( *Bacillus* sp.) ^16^, and the carbon source was from starters based on the tested treatments with the *C*/ *N* ratio of 20:1. The starter was applied in cultivation media and probiotic bacteria administration. Probiotics and starter were applied into cultivation cultured media and left for 1 week, and the media were aerated continuously. The starters were added every week for maintaining the floc. Floc formation was indicated by green water and foam forming in the media.

The number of fish used in this study was 360 individuals and they were obtained from a hatchery in the Bengkalis Regency, Riau. The fish were 50 days old, 3.71±0.11 cm TL. As the sex of the fish cannot be identified morphologically, there is no separation of fish sex in this study. The fish was distributed into research tanks as the flocs are formed, 7 days after the starter was cultured in the tanks (total 12 tanks, 100L volume). The density of the fish was 30 fishes/100 L. Stocking of red tilapia larvae in the morning at 8 AM with a water temperature of 25°C The fish were fed on commercial diet containing 38% crude protein, 5% crude lipid, and 6% crude fiber (PF1000, PT Matahari Sakti). Feeding was conducted *ad libitum*/satiation, 2 times/day (8 AM and 5 PM) for 40 days.

### Measurement of nitrogen content

The ammonia (NH _3_), nitrite (NO _2_^−^), and nitrate (NO _3_^+^) contents were measured by spectrophotometry. These parameters were measured every 8 days in each treatment for 40 days. Ammonia (NH _3_), Nitrite (NO _2_^−^) and Nitrate (NO _3_^+^) were measured using spectrophotometer (Optima - SP300) at a wavelength of 630 nm ^27^. The measurement was conducted at 8:00 AM with three replications at 8-day intervals over 40 days (six timepoints).

### Statistical analysis

The data were subjected to one-way analysis of variance followed by the post hoc Newman–Keuls test using SPSS 18.0 software. P<0.05 was considered to indicate a statistically significant difference.

## Results

Over the six timepoints measured, the mean concentration of ammonia ranged from 0.02 mg L ^−1^ to 0.07 mg L ^−1^ (with an average value of 0.03 ± 0.02 mg L ^−1^), nitrite ranged from 0.20 mg L ^−1^ to 0.43 mg L ^−1^ (with an average value of 0.25 ± 0.12 mg L ^−1^), and nitrate ranged from 0.64 mgL ^−1^ to 3.20 mgL ^−1^ (with an average value of 1.42 ± 1.19 mg L ^−1^). The study revealed that the best concentrations of ammonia, nitrite, and nitrate were recorded in the biofloc starter of molasses. These values did not significantly differ from those obtained with the tapioca and sucrose starters, but they were significantly different from the control treatment without a starter ( Table 1). Raw values for each replicate are available as *Underlying data* ^28^.

**Table 1.** Average values of ammonia nitrogen, nitrite, nitrate, turbidity, and carbon dioxide concentrations based on treatment. Data values are the mean and standard deviation. Mean values with different superscripts in the same row were significantly different ( *p* < 0.05).

Parameter	Unit	Treatment			
		Control (A)	Molasses (B)	Tapioca (C)	Sucrose (D)
NH _3_-N	mgL ^−1^	0.07 ± 0.05 ^b^	0.02 ± 0.01 ^a^	0.02 ± 0.01 ^a^	0.02 ± 0.01 ^a^
NO _2_-N	mgL ^−1^	0.43 ± 0.25 ^b^	0.14 ± 0.09 ^a^	0.20 ± 0.12 ^a^	0.24 ± 0.14 ^a^
NO _3_-N	mgL ^−1^	3.54 ± 3.21 ^b^	0.64 ± 0.46 ^a^	0.90 ± 0.96 ^a^	0.93 ± 1.12 ^a^

**Figure 1.**
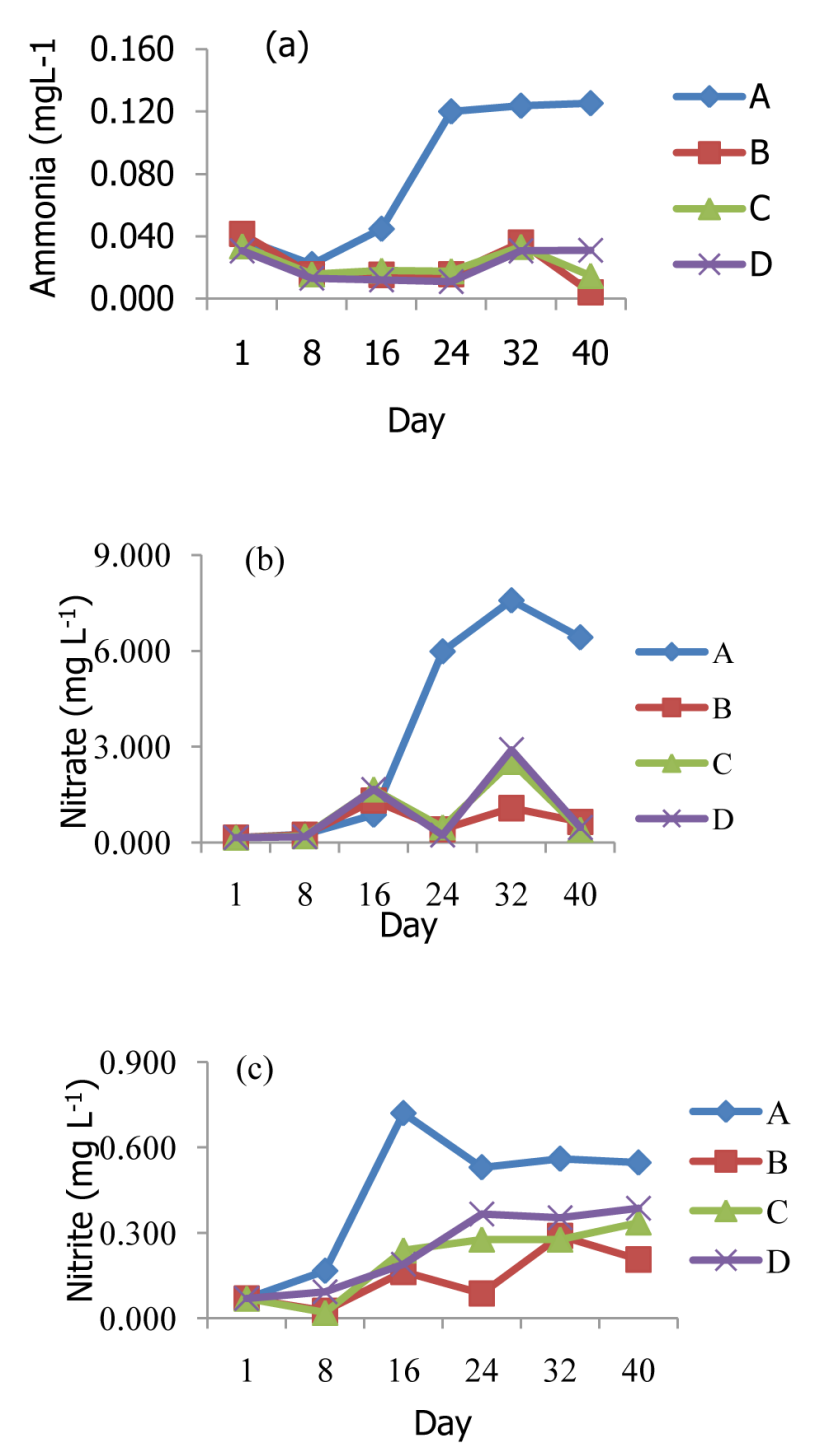
( **a**) Concentrations of ammonia, ( **b**) nitrate, and ( **c**) nitrate during 40 days of the experiment. A = control treatment, B = molasses starter, C = tapioca starter, and D = sucrose starter.

In the control treatment, the concentrations of ammonia, nitrite, and nitrate increased gradually with increasing experimental time. However, in the starter treatments, the ammonia concentration dropped during the first week of the experiment (day 8), became stagnant during the second and third weeks (day 16 to day 24), and increased again at day 32 of the experiment. However, the ammonia concentration decreased sharply at day 40 ( Figure 1a). The nitrite concentration also decreased during the first week but increased slightly at day 16 and increased gradually until day 40 in treatments C (tapioca) and D (sucrose). By contrast, the nitrite concentration decreased at day 24, increased at day 32, and decreased at day 40 ( Figure 1b). The nitrate concentration was relatively stagnant from day 1 to day 8. The nitrate concentration fluctuated during the experiment ( Figure 1c). In general, the molasses starter (treatment B) yielded slightly better results than the other starters, but no significant different was found between the treatments except the control. The data of the growth performance of the red tilapia fish *O. niloticus* has been published separately ^29^.

## Discussion

The study revealed that the concentrations of ammonia, nitrite, and nitrate were significantly lower in the biofloc system using starters compared with those in the system without a starter (control). Biofloc has probiotic bacteria that can change ammonia to nontoxic materials (such as nitrate) that are useful for phytoplankton growth. Therefore, the ammonia and nitrate concentrations are low in the culture media ^19, 21, 30^. Biofloc does not necessarily only contain bacteria (for example, *Bacillus*), but is also composed of other useful microorganisms such as microalgae and zooplankton that are trapped by organic particles ^31^. Algae and zooplankton can be used by cultured biota (tilapia) as natural food.

In general, the starters used in this study were carbohydrate compounds. However, the study showed that the molasses starter yielded slightly better results compared with the other starters. This finding indicated that molasses was the best carbon source for biofloc in the tilapia culture system. Molasses can provide a sufficient carbon level for heterotrophic bacteria that use this carbon as an energy source for growth ^6, 19, 21, 23, 32^. Molasses are a liquid byproduct from the sugar industry. This material has a total carbon content of around 37% ^24^. Therefore, molasses are rapidly soluble in water and can be quickly absorbed by heterotrophic bacteria ^3^. In terms of chemical structure, molasses are classified as a simple carbohydrate containing six C atoms (monosaccharides), while sucrose (treatment D) is a combination of two monosaccharides that contain 12 C atoms (sucrose). Tapioca is classified as a complex carbohydrate (60,000 C atoms) and is more slowly digested by bacteria than molasses ^25, 33^.

## Conclusion

Carbon source from molasses is effective in reducing concentrations of ammonia, nitrite, and nitrate in red tilapia culture with biofloc technology.

## Data availability

### Underlying data

Figshare: Effect of different biofloc starters on ammonia, nitrate, and nitrite concentrations in the cultured tilapia Oreochromis niloticus system. https://doi.org/10.6084/m9.figshare.12027951 ^28^.

This project contains raw data for the concentration of ammonia, nitrate and nitrite in each tank at each timepoint.

Data are available under the terms of the Creative Commons Attribution 4.0 International license (CC-BY 4.0).

## References

[1] Emerenciano MGC Córdova LRM Porchas MM: Biofloc Technology (BFT): A Tool for Water Quality Management in Aquaculture. *Water Qual.* 2017;5:92–109. 10.5772/66416

[2] Putra I: Effectiveness of Nitrogen absorption with different Filter Medium in Tilapia ( *Oreochromis niloticus*) cultured in the recirculation system. Bogor Agriculture University.2010 Reference Source

[3] Serra FP Goana CAP Furtado PS: Use of different carbon sources for the biofloc system adopted during the nursery and grow-out culture of *Litopenaeus vannamei.* *Aquac Int.* 2015;23(6);1325–1339. 10.1007/s10499-015-9887-6

[4] De Schryver PD Verstraete W: Nitrogen removal from aquaculture pond water by heterotrophic nitrogen assimilation in lab-scale sequencing batch reactors. *Bioresour Technol.* 2009;100:1162–1167. 10.1016/j.biortech.2008.08.043 18842400

[5] Avnimelech Y: Carbon Nitrogen Ratio as a Control Element in Aquaculture Systems. *Aquaculture.* 1999;176(3–4):227–235. 10.1016/S0044-8486(99)00085-X

[6] Azim ME Little DC: The biofloc technology (BFT) in indoor tanks: water quality, biofloc composition, and growth and welfare of Nile tilapia ( *Oreochromis niloticus*). *Aquaculture.* 2008;283(1–4):29–35. 10.1016/j.aquaculture.2008.06.036

[7] Djokosetiyanto D Sunarma A Widanarni: Changes of Ammonia, Nitrite and Nitrate at Recirculation System of Red Tilapia ( *Oreochromis.* sp.) Rearing. *Jurnal Akuakultur Indonesia.* 2006;5(1):13–20. 10.19027/jai.5.13-20

[8] Stein LY Klotz MG: The nitrogen cycle. *Curr Biol.* 2016;26(3):R94–98. 10.1016/j.cub.2015.12.021 26859274

[9] Jiménez-Ojeda YK Collazoz-Lasso LF Arias-Castellanos JA: Dynamics and use of nitrogen in Biofloc Technology – BFT. *AACL Bioflux.* 2018;11(4):1107–1129. Reference Source

[10] Ebeling JM Timmons MB Bisogni JJ: Engineering analysis of the stoichiometry of photoautotrophic, autotrophic and heterotrophic removal of ammonia–nitrogen in aquaculture systems. *Aquaculture.* 2006;257(1–4):346–358. 10.1016/j.aquaculture.2006.03.019

[11] Zulfahmi I Syahimi M Muliari: Influence of Biofloc Addition With Different Dosages on The Growth of Tiger Shrimp Juvenile ( *Penaeus monodon.* Fabricius 1798). *J Biol.* 2018;11(1):1–8. 10.15408/kauniyah.v11i1.4862

[12] Nurhatijah N Muchlisin ZA Sarong MA: Application of biofloc to maintain the water quality in the culture system of the tiger prawn ( *Penaeus monodon*). *AACL Bioflux.* 2016;9(4):923–928. Reference Source

[13] Supriatna A Nurhatijah H Sarong MA: Effect of biofloc density and crude protein level in the diet on the growth performance, survival rate, and feed conversion ratio of Black Tiger Prawn ( *Penaeus monodon*). *IOP Conf. Series: Earth and Environmental Science* 2019;348:012131 10.1088/1755-1315/348/1/012131

[14] Avnimelech Y: Control of Microbial Activity in Aquaculture Systems: Aktive Suspension Ponds. *Biofloc Technology A Practical Guide Book, 2 edition.*United States: The World Aquaculture Society.2012;34(4):19–21. Reference Source

[15] Ombong F Indra RNS: Application of Biofloc Technology (BFT) in Tilapia ( *Oreochromis niloticus*) Culture. *Jurnal Akuakultur Rawa Indonesia.* 2016;4(2):16–25[in Indonesian].

[16] Putra I Rusliadi, Fauzi M: Growth performance and feed utilization of African catfish *Clarias gariepinus* fed a commercial diet and reared in the biofloc system enhanced with probiotic [version 1; peer review: 2 approved]. *F1000Res.* 2017;6:1545. 10.12688/f1000research.12438.1 28944046 PMC5585874

[17] Taw N: Shrimp Farming in Biofloc System, Review and recent developments. FAO project, Blue Archipelago. Presented in World Aquaculture. Adelaide.2014 Reference Source

[18] Rohmana D: Conversion of catfish farming waste, Clarias sp. into heterotrophic bacterial biomass for improving the quality of water and food of giant prawns, *Macrobrachium rosenbergii* . Bogor Agriculture University. Bogor.2009;64 [ *in*Indonesian]. Reference Source

[19] De Schryver P Crab R Defoirdt T: The Basic of Bioflocs Technology, The Added Value for Aquaculture.2008;277(3–4):125–137. 10.1016/j.aquaculture.2008.02.019

[20] De Lima PCM da Silva LOB Abreu JDL: Tilapia Cultivated in A Low-Salinity Biofloc System Supplemented with Chlorella vulgaris and Differents Molasess Application Rates. *Boletim do Instituto de Pesca.* 2019;45(4):e494 10.20950/1678-2305.2019.45.4.494

[21] Hargreaves JA: Photosynthetic suspended-growth systems in aquaculture. *Aquaculture Engineering.* 2006;34(3):344–363. 10.1016/j.aquaeng.2005.08.009

[22] Suwoyo HS Abdul M Gunarto G: Use of Organic Carbon Sources in the culture of Vaname Shrimp ( *Litopenaeus vannamei*) with Biofloc Technology. *Indoaqua Proceeding. Aquaculture Technology Innovation Forum* 2012 [in Indonesian]. Reference Source

[23] Avnimelech Y: BioflocTechnology, A Practical Guide Book. The World Aquaculture Society. Louisiana, United States2009;120 Reference Source

[24] Suastuti NGAMDA: Utilization of By-Products of the Agriculture Industry Molasses and Tofu Liquid Waste as a Source of Carbon and Nitrogen for Biosurfactant Production by Bacillus sp Commercial Strain and Workshop. Bogor Agricultural University. Bogor.1998;105 [ *in Indonesia*]. Reference Source

[25] Azhar MH: The Role of External Carbon Resources Different in Biofloc Formation and Its Effect on Water Quality and Production in the Vanamme Shrimp Culture System. Bogor Agricultural Institute,2013;39 [in Indonesian]. Reference Source

[26] Purnomo PD: The Effect quaculture Media towards Production of Intensive Tilapia Culture ( *Oreochromis niloticus*). faculty of Fisheries and Marine Science, Universitas Diponegoro.2012:1:161–179. Reference Source

[27] APHA (American Public Health Assocition): Standard methoda for the examinatation of water and wastewater. 23RD. Edition.1989 Reference Source

[28] Putra I Effendi I Lukistyowati I: Effect of different biofloc starters on ammonia, nitrate, and nitrite concentrations in the cultured tilapia Oreochromis niloticus system. *figshare.*Dataset.2020 10.6084/m9.figshare.12027951.v1 PMC7241270 32509278

[29] Putra I Effendi I Lukistyowati I: Growth and survival rate of red tilapia ( *Oreochromis* sp.) cultivated in the brackish water tank under biofloc system. *Adv Eng Res.* 2019;90:96–99. 10.2991/iccelst-st-19.2019.19

[30] Crab R Avnimelech Y Defoirdt T: Nitrogen Removal Techniques in Aquaculture for Sustainable Production. *Aquaculture.* 2007;270(1–4):1–14. 10.1016/j.aquaculture.2007.05.006

[31] Hastuti S Subandiyono S: Production Performance of African Catfish ( *Clarias gariepinus,* burch) were Rearing with Biofloc technology. *J Fish Sci Technol.* 2014;10(1):37–42. Reference Source

[32] Ray AJ Kevin SD Jeffrey ML: Water Quality Dynamics and Shrimp ( *Litopenaeus vannamei*) Production in Intensive, Mesohaline Culture System With Two levels of Biofloc Management. *Aquaculture Engineering.* 2011;45(3):127–136. 10.1016/j.aquaeng.2011.09.001

[33] Chamberlain G Avnimelech Y McIntosh RP: Advantages of aerated microbial reuse systems with balanced C/N. Nutrient transformation and water quality benefits. *Global Aquaculture Alliance Advocate.* 2001;4:53–56. Reference Source

