# Supplementary material for: Effect of different biofloc starters on ammonia, nitrate, and nitrite concentrations in the cultured tilapia Oreochromis niloticus system
Source: F1000Res. 2020 Jun 2;9:293. Originally published 2020 Apr 24. [Version 3] doi: 10.12688/f1000research.22977.3 (PMC7241270; doi:10.12688/f1000research.22977.3)
Supplement: Peer review history

# Peer review: Reviewer response for version 3

**DOI:** 10.5256/f1000research.27000.r64225

**Published:** 2020-06-03

**Authors:** Muhammad Rizal Razman

**Affiliations:** 1 Research Centre for Sustainability Science and Governance (SGK), Institute for Environment and Development (LESTARI), Universiti Kebangsaan Malaysia (UKM), Bangi, Malaysia

The paper has been amended accordingly as suggested.

Is the work clearly and accurately presented and does it cite the current literature?

Yes

If applicable, is the statistical analysis and its interpretation appropriate?

Yes

Are all the source data underlying the results available to ensure full reproducibility?

Yes

Is the study design appropriate and is the work technically sound?

Yes

Are the conclusions drawn adequately supported by the results?

Partly

Are sufficient details of methods and analysis provided to allow replication by others?

Yes

Reviewer Expertise:

Agricultural and Biological Sciences, Environmental Science, Sustainability Science and Governance for Sustainablity

I confirm that I have read this submission and believe that I have an appropriate level of expertise to confirm that it is of an acceptable scientific standard.

# Peer review: Reviewer response for version 3

**DOI:** 10.5256/f1000research.27000.r64226

**Published:** 2020-06-03

**Authors:** Christopher Marlowe A. Caipang

**Affiliations:** 1 University of San Agustin, Iloilo City, Philippines

The authors have addressed the suggestions/comments made on the manuscript.

Is the work clearly and accurately presented and does it cite the current literature?

Partly

If applicable, is the statistical analysis and its interpretation appropriate?

Partly

Are all the source data underlying the results available to ensure full reproducibility?

Yes

Is the study design appropriate and is the work technically sound?

Yes

Are the conclusions drawn adequately supported by the results?

Partly

Are sufficient details of methods and analysis provided to allow replication by others?

Partly

Reviewer Expertise:

Aquaculture, biotechnology, aquatic microbiology, fish health management

I confirm that I have read this submission and believe that I have an appropriate level of expertise to confirm that it is of an acceptable scientific standard.

# Peer review: Reviewer response for version 2

**DOI:** 10.5256/f1000research.26350.r63360

**Published:** 2020-05-20

**Authors:** Christopher Marlowe A. Caipang

**Affiliations:** 1 University of San Agustin, Iloilo City, Philippines

The authors have addressed the issues and suggestions in the earlier draft. However, I have notice that the statement in the revised version "Presently, no information is available on the best starter for biofloc in the cultured system of Nile tilapia *Oreochromis niloticus*." is still there and no additional information was given. The authors must indicate a thorough literature search if indeed no best starter is currently available.

 If the authors can address this issue, then the manuscript can be considered to be accepted and indexed in the journal.

Is the work clearly and accurately presented and does it cite the current literature?

Partly

If applicable, is the statistical analysis and its interpretation appropriate?

Partly

Are all the source data underlying the results available to ensure full reproducibility?

Yes

Is the study design appropriate and is the work technically sound?

Yes

Are the conclusions drawn adequately supported by the results?

Partly

Are sufficient details of methods and analysis provided to allow replication by others?

Partly

Reviewer Expertise:

Aquaculture, biotechnology, aquatic microbiology, fish health management

I confirm that I have read this submission and believe that I have an appropriate level of expertise to confirm that it is of an acceptable scientific standard.

# Peer review: Reviewer response for version 1

**DOI:** 10.5256/f1000research.25366.r62751

**Published:** 2020-05-11

**Authors:** Muhammad Rizal Razman

**Affiliations:** 1 Research Centre for Sustainability Science and Governance (SGK), Institute for Environment and Development (LESTARI), Universiti Kebangsaan Malaysia (UKM), Bangi, Malaysia

1. The work was clearly and accurately presented. 
2. The work has cited the current literature, (36 %) of (2015-2020).
3. The study design was appropriate and  the work  was technically sound.
4. The work presented sufficient details of methods and analysis provided to allow replication by others.
5. The statistical analysis and its interpretation was appropriate.
6. The source of data underlying the results available to ensure full reproducible.
7. The authors should elaborate more for their conclusion. The conclusion should be reflecting the work that has been done. The conclusion given was too brief.

Is the work clearly and accurately presented and does it cite the current literature?

Yes

If applicable, is the statistical analysis and its interpretation appropriate?

Yes

Are all the source data underlying the results available to ensure full reproducibility?

Yes

Is the study design appropriate and is the work technically sound?

Yes

Are the conclusions drawn adequately supported by the results?

Partly

Are sufficient details of methods and analysis provided to allow replication by others?

Yes

Reviewer Expertise:

(Agriculture and Biological Sciences, Environmental Science, Sustainability Science and Governance, Environment and Development)

I confirm that I have read this submission and believe that I have an appropriate level of expertise to confirm that it is of an acceptable scientific standard.

# Peer review: Reviewer response for version 1

**DOI:** 10.5256/f1000research.25366.r62750

**Published:** 2020-05-01

**Authors:** Christopher Marlowe A. Caipang

**Affiliations:** 1 University of San Agustin, Iloilo City, Philippines

Summary: This ms aimed to determine  the best starter of biofloc in a red tilapia *Oreochromis niloticus* culture system. Three biofloc starters (molasses, tapioca and sucrose) and control were tested in triplicate over a period of 40 days. Nitrate, nitrite and ammonia were monitored during the study. Based on the findings, molasses is considered to be the best starter for biofloc production among the starters that were tested.

 Comments: The work presented the findings in a straightforward manner. However, the ms can be further improved following these suggestions:

1. Change the title to: Effect of different biofloc starters on ammonia, nitrate, and nitrite concentrations in the culture of tilapia *Oreochromis niloticus*.
2. The authors mentioned red tilapia in the Abstract but only tilapia in other portions of the text. Please be consistent.
3. In the Introduction, the authors wrote: "Molasses, tapioca, and wheat flours are common starters in biofloc culture". Please indicate to which species were these starters used.
4. The authors wrote: "Presently, no information is available on the best starter for biofloc in the cultured system of Nile tilapia *Oreochromis niloticus*". It is suggested that the authors conduct a thorough literature search, as there have been studies done on biofloc starters in tilapia. Please rectify this statement.
5. In the methods, please indicate how the biofloc was maintained in the study. The study only made mentioned the starter. In the 40 days, was there no addition of the starters to maintain the biofloc?
6. Please indicate water exchange rates? There was no water change in the control in 40 days, and the fish were quite big during stocking (approximately 3 g). With no water exchange in 40 days in the control and with the addition of feed, there is definitely a deterioration of water quality and lower fish survival. In the actual culture of tilapia, water exchange is done. For biofloc system, water exchange is minimal hence the results are compared in terms of water quality as well as savings in the cost of electricity or volume of water discharge.
7. Was feeding the same for the biofloc treatments and control? Please explain.
8. The authors should explain the water exchange and feeding management between the control and biofloc treatments and how these are controlled/managed relative to the study objectives.
9. Perform a correlation analysis on the water quality parameter over time. Related the values of the slope and intercept on the effects of water quality for each treatment.
10. ANOVA should also be done on the water quality per sampling point so that the readers will know when the differences became significant.
11. Why did the authors conclude that molasses is the best starter? Based on the graph, all three starters had better water quality than the control. In addition, the differences in the water quality values (ammonia, nitrite, nitrate) among the biofloc treatments were not significantly different. It is suggested that authors perform additional statistical test to show that molasses is significantly better than the other 2 biofloc starters.
12. The role of the probiotics in the study should be discussed. The authors should provide in the methods the initial counts of the probiotics that were applied initially.

I hope that these suggestions will be considered by the authors when they revise the manuscript.

Is the work clearly and accurately presented and does it cite the current literature?

Partly

If applicable, is the statistical analysis and its interpretation appropriate?

Partly

Are all the source data underlying the results available to ensure full reproducibility?

Yes

Is the study design appropriate and is the work technically sound?

Yes

Are the conclusions drawn adequately supported by the results?

Partly

Are sufficient details of methods and analysis provided to allow replication by others?

Partly

Reviewer Expertise:

Aquaculture, biotechnology, aquatic microbiology, fish health management

I confirm that I have read this submission and believe that I have an appropriate level of expertise to confirm that it is of an acceptable scientific standard, however I have significant reservations, as outlined above.

# Response

**Published:** 2020-05-04

**Authors:** Iskandar Putra

**Affiliations:** University of Riau, Indonesia

1. Inquiry 

 Change the title to: Effect of different biofloc starters on ammonia, nitrate, and nitrite concentrations in the culture of tilapia *Oreochromis niloticus*.

 Response: Thank you for your suggestion. I have already changed it

 2. Inquiry

 The authors mentioned red tilapia in the Abstract but only tilapia in other portions of the text. Please be consistent.

 Response: Thank you for your suggestion. I have already changed it

 3. Inquiry

 In the Introduction, the authors wrote: "Molasses, tapioca, and wheat flours are common starters in biofloc culture". Please indicate to which species were these starters used.

 Response: We added this information in Introduction

 4. Inquiry

 The authors wrote: "Presently, no information is available on the best starter for biofloc in the cultured system of Nile tilapia *Oreochromis niloticus*". It is suggested that the authors conduct a thorough literature search, as there have been studies done on biofloc starters in tilapia. Please rectify this statement.

 Response: We have modified this statement

 5. Inquiry

 In the methods, please indicate how the biofloc was maintained in the study. The study only made mentioned the starter. In the 40 days, was there no addition of the starters to maintain the biofloc?

 Response: The starters were added every week for maintaining the floc

 6. Inquiry

 Please indicate water exchange rates? There was no water change in the control in 40 days, and the fish were quite big during stocking (approximately 3 g). With no water exchange in 40 days in the control and with the addition of feed, there is definitely a deterioration of water quality and lower fish survival. In the actual culture of tilapia, water exchange is done. For biofloc system, water exchange is minimal hence the results are compared in terms of water quality as well as savings in the cost of electricity or volume of water discharge.

 Response: There is no water exchange during conducting the experiment

 7. Inquiry

 Was feeding the same for the biofloc treatments and control? Please explain.

 Response: Feeding applied for the biofloc treatments and control were same

 8. Inquiry

 The authors should explain the water exchange and feeding management between the control and biofloc treatments and how these are controlled/managed relative to the study objectives.

 Response: There was no water exchange during the experiment and feeding was applied the same treatment for the biofloc treatments and control.

 9. Inquiry

 Perform a correlation analysis on the water quality parameter over time. Related the values of the slope and intercept on the effects of water quality for each treatment.

 Response: We did not perform a correlation analysis on the water quality parameter over time.

 10. Inquiry

 ANOVA should also be done on the water quality per sampling point so that the readers will know when the differences became significant.

 Response: We calculated ANOVA of the water quality according to data at the end of the experiment

 11. Inquiry

 Why did the authors conclude that molasses is the best starter? Based on the graph, all three starters had better water quality than the control. In addition, the differences in the water quality values (ammonia, nitrite, nitrate) among the biofloc treatments were not significantly different. It is suggested that authors perform additional statistical test to show that molasses is significantly better than the other

 2 biofloc starters.

 Response: Based on the ANOVA, there was no significant difference of water quality among the starter treatments, but the molasses supplementation in the culture media showed the best water quality values compared to other starters

 12. Inquiry

 The role of the probiotics in the study should be discussed. The authors should provide in the methods the initial counts of the probiotics that were applied initially.

 Response: Thank you for your suggestion. We added this information in methods
